# Gene target selection for loop-mediated isothermal amplification for rapid discrimination of *Treponema pallidum* subspecies

**DOI:** 10.1371/journal.pntd.0006396

**Published:** 2018-04-12

**Authors:** Sascha Knauf, Simone Lüert, David Šmajs, Michal Strouhal, Idrissa S. Chuma, Sieghard Frischmann, Mohammed Bakheit

**Affiliations:** 1 Work Group Neglected Tropical Diseases, Infection Biology Unit, German Primate Center, Leibniz- Institute for Primate Research, Göttingen, Germany; 2 Department of Biology, Faculty of Medicine, Masaryk University, Brno, Czech Republic; 3 Sokoine University of Agriculture, Faculty of Veterinary Medicine, Morogoro, Tanzania; 4 Mast Diagnostica GmbH, Reinfeld, Germany; Christian Medical College, Vellore, INDIA

## Abstract

We show proof of concept for gene targets (*polA*, *tprL*, and TP_0619) that can be used in loop-mediated isothermal amplification (LAMP) assays to rapidly differentiate infection with any of the three *Treponema pallidum* subspecies (*pallidum* (*TPA*), *pertenue* (*TPE*), and *endemicum* (*TEN*)) and which are known to infect humans and nonhuman primates (NHPs). Four *TPA*, six human, and two NHP *TPE* strains, as well as two human *TEN* strains were used to establish and validate the LAMP assays. All three LAMP assays were highly specific for the target DNA. Amplification was rapid (5–15 min) and within a range of 10E+6 to 10E+2 of target DNA molecules. Performance in NHP clinical samples was similar to the one seen in human *TPE* strains. The newly designed LAMP assays provide proof of concept for a diagnostic tool that enhances yaws clinical diagnosis. It is highly specific for the target DNA and does not require expensive laboratory equipment. Test results can potentially be interpreted with the naked eye, which makes it suitable for the use in remote clinical settings.

## Author summary

Sustainable eradication of human yaws benefits from applicable and reliable assays to detect possible reemerging yaws cases. Our study provides proof of concept for LAMP assays that are capable of rapid diagnoses and discrimination of active *Treponema pallidum* infection. While current clinical diagnosis is based on the clinical manifestations in combination with serology, the selected targets and LAMP assays allow for DNA based differentiation between skin ulcers caused by the subsp. *pallidum* (syphilis), subsp. *pertenue* (yaws), and the subsp. *endemicum* (bejel). The presented LAMP assays require limited expensive technical equipment and can be run in virtually any clinical setting. The method is thus capable of enhancing yaws diagnosis in particular in a low capacity environment.

## Introduction

Human yaws is a tropical skin disease of children caused by the bacterium *Treponema pallidum* subsp. *pertenue* (*TPE*) [1]. Skin ulcers are the most characteristic clinical manifestations associated with infection in all three active disease stages (primary, secondary, and tertiary yaws) [2]. The disease is currently subject to global eradication efforts [3], which face challenges that arise from the biology and distribution of the yaws bacterium as well as its diagnosis and treatment [4]. It is largely believed that the first yaws eradication campaigns conducted in the mid-1950s to late 1960s were successful in terms of reducing the prevalence by 95% but failed to eradicate the disease when local efforts to prevent new cases proved insufficient [5]. The majority of affected populations belong to poor and marginalized societies, with only rudimentary access to health care systems (‘Where the road ends, yaws begins’) [6]. Until today, standard diagnosis of yaws in clinical settings is based on clinical manifestations in combination with serology [1]. *T*. *pallidum* (*TP*) elicits a strong antibody response [7, 8]. Although it is possible to distinguish current infection (active or latent) from past infection when non-treponemal and treponemal tests are used in combination [9], it remains impossible based on serology and in some instances clinical manifestations, to differentiate yaws infection (*TPE*) from syphilis (caused by subsp. *pallidum* (*TPA*)) or bejel (caused by the subsp. *endemicum* (*TEN*)). Moreover, it has been shown that other diseases are capable of mimicking yaws infection. In particular, *Haemophilus ducreyi* has been reported to cause yaws-like skin ulcers [10]. Lastly, a larger number of skin ulcers in rural Africa remains etiologically undiagnosed [11], which increases the chance of overlooked infection with *TPE*. Other diseases which are capable of mimicking yaws infection are cutaneous leishmaniasis, scabies, or fungal infections [1]. Eradication of yaws is further challenged by the finding that nonhuman primates (NHPs) are infected with *TP* [12, 13]. Notably, all whole genome sequenced simian strains must be considered *TPE* strains [14, 15]. NHPs therefore must be considered as a possible natural reservoir for human infection [13]. The West African simian *TPE* strain Fribourg-Blanc, which was isolated from a Guinea baboon (*Papio papio*) in the 1960s [16], caused sustainable infection when inoculated into humans [17].

Post-eradication surveillance following the currently ongoing mass-azithromycin treatment phase [4] would benefit from rapid and cost-effective molecular tests that are able to distinguish *TPE* infection [18] from infections with all other *TP* subspecies (*TPA* and *TEN*) and bacteria that are involved in tropical skin ulcers and which may fall together with *TP* seropositivity. Potentially a single overlooked yaws case would result in a failure of global yaws eradication. Loop-mediated isothermal amplification (LAMP) was first described by Notomi et al. in 2000 [19] and since then has been extensively used to improve infectious disease diagnostics [20]. The highly specific method recognizes the DNA target using six distinct sequences initially and four distinct sequences subsequently [19]. LAMP uses a DNA polymerase with high strand displacement activity to perform a fast running auto-cycling strand displacement synthesis. Reactions run at constant temperature (isothermal) and therefore do not require expensive technical equipment such as PCR cycling machines. Our objective was to identify suitable gene targets that can be used for LAMP assay design to rapidly distinguish between yaws infection, including simian strains, and syphilis or bejel.

## Materials and methods

### Ethical statement and *TP* strain selection

DNA samples of human *TPA* laboratory strain Mexico A, Nichols, Seattle 81–4, SS14, *TPE* strain Gauthier, CDC-1, CDC-2, Samoa D, Sei Geringging K403, Kampung Dalan K363, as well as the simian *TPE* strain Fribourg-Blanc were obtained from rabbit-in vivo inoculation experiments (S.A. Lukehart and DS). These experiments were not directly associated with this study. DNA extracts from human *TEN* strain Bosnia A and Iraq B originate from whole genome amplified clinical samples (DS) that were not directly associated with this study. DNA from a *TP*-infected olive baboon (*Papio anubis*; 6RUM2090716) originates from a clinical sample collected for a different study at Ruaha National Park (RNP) in Tanzania in 2015 (DFG KN1097/3-1 (SK)). Details and further reference for each strain included into the study can be found in the Supplementary S1 Table. ‘Good Veterinary Practice’ rules were applied to all procedures where animals were handled.

### Study design and data collection

Three different LAMP assays were designed. First, we generated a LAMP assay that is able to detect DNA of all three *TP* subspecies (*TPA*, *TPE*, and *TEN*). This assay served as an initial control and was designed for the use in NHPs where little is known about the *TP* subspecies that circulate in wild NHP populations. Second, a LAMP assay was designed to distinguish *TPE* strain infection from infection with *TPA* or *TEN* strains. Third, a LAMP assay that differentiates between infection with *TPE* or *TEN* and infection with *TPA* strains has been established. All LAMP reactions were run with four human *TPA*, six human *TPE* and two simian *TPE* strains, as well as two human *TEN* strains of known copy number (S1 Table). All tests were run as triplicates and included a DNA-free negative control.

Dilution series of target DNA were used to identify the analytic limits of detection for each of the specific LAMP reactions using appropriate strain material. 10-fold serial dilutions of the target DNA were applied to cover a range of at least five decimal powers, from the maximum of *TP* copy numbers (strain Nichols 10E+5, all other strains 10E+6) until 10E+0. Negative controls that contained no DNA and dilution steps that contained ≤10E+2 *TP* copies were run as at least six replicates. A StepOnePlus Real-Time PCR System (ThermoFisher Scientific) was used to run the reactions and to collect the data. Due to software restrictions, it was necessary to introduce a (neglectable) thermal cycling step into the protocol. Each LAMP run therefore encompassed continuous 40 cycling steps each consistent of 63°C for five seconds followed by 64°C for one minute and data collection.

### LAMP reactions

LAMP reactions were performed in a volume of 25.0 μl using the Mast Isoplex DNA Kit (#REF67dnamp, Mast Diagnostica GmbH). According to the manufacture’s guidance, each reaction consisted of 12.5 μl of the kit’s 2x reaction mix, 1.0 μl Bst polymerase enzyme, 1.0 μl fluorochrome dye, and 2.0 μl of the primer mix. One microliter target DNA was included and distilled water (molecular grade) was used to top up the reaction volume until 25.0 μl were reached. All primers were heat pre-treated at 95°C for 5 min and immediately cooled on ice prior to adding them to the master mix. The primer mix contained 1.6 μM each FIP and BIP, 0.2 μM each F3 and B3, as well as 0.8 μM each LF and LB primer. All reactions were run on a MircoAmp Fast Optical 96-well reaction plate (#4346907, ThermoFisher).

### Oligonucleotide primer design

Oligonucleotide primers were designed using the PrimerExplorer v5 Software (http://primerexplorer.jp/e/). Each LAMP primer set consisted of six oligonucleotide primers (Table 1). The design followed the description given by Yoshida et al. 2005 [21]. Briefly, a set of four primers (F3, B3, the forward inner primer [FIP], and backward inner primer [BIP]), which bind six loci of the target gene (F1, F2, F3, B1, B2, and B3) are necessary. The two inner primers (FIP and BIP) are a sequence combination of sense and antisense sequences of the DNA. This is essential for the priming in the first stage and the self-priming in the later stages. Therefore, FIP primers consist of the combination of sequences defined as F1c (c = complementary) and F2. Likewise, BIP primers are composed of primer sequences B1c and B2. To enhance amplification efficacy, two loop primers LF and LB were added to each of the LAMP primer sets. To confirm the specificity of the newly designed primers, we performed a search for orthologous sequences using BLASTn at the NCBI homepage (http://blast.ncbi.nlm.nih.gov/Blast.cgi).

**Table 1:** Oligonucleotide primers used in this study.

LAMP	Type	Sequence	Gene target
*TP*	F3	5’-ATTGGTCCTAAGACGGCT	*polA* (TP_0105)
	B3	5’-GCGGAATACAACAGGAATC	
	FIP	5’-CAGCGCTTCTTTTAAGGAATAGGTAGCACATCTTCTCCACTGT	
	BIP	5’-CGCACGAAGATAGTGTGTGGACATGGTACATCGTCACG	
	LF	5’-CGATAAATACCATCAAGTGTGCCAAA	
	LB	5’-GAAGAAAGATGCATTTTTTTCTCGTTC	
*TPA*/*TEN*	F3	5’-TGCAGTCTTTTTTTGTGCGG	*tprL* (TP_1031)
	B3	5’-TCAAATCATTGGTGGTGCGA	
	FIP	5’-CTGCGGGGCAATGCCTAGCTGCTCCCGGGGTTTGG	
	BIP	5’-GTAACTGGTCACGCCCAGCTGCCCGTGCGTGTACTCGTTC	
	LF	5’-AGAAAAAACGGGCAGTGCG	
	LB	5’-GGGGCATTAAGTTTAAAAAGAACCC	
*TPE*/*TEN*	F3	5’-TCCCTCGAGACTCGATTCC	TP_0619
	B3	5’-GTACGCATCTGTCGGTAGC	
	FIP	5’-GTTGCACAGAAGGGGGGTGGCATCCTTCTGatGCGTCAGC	
	BIP	5’-GTGCTTTTCGGGAGGATTCCCGAGTGCACTGCGCTCATCT	
	LF	5’-TTCCGCGTTCGGTGCTC	
	LB	5’-CTTTCGGGACGATGAGATAATGC	

### Gene locus selection

**Fig 1:**
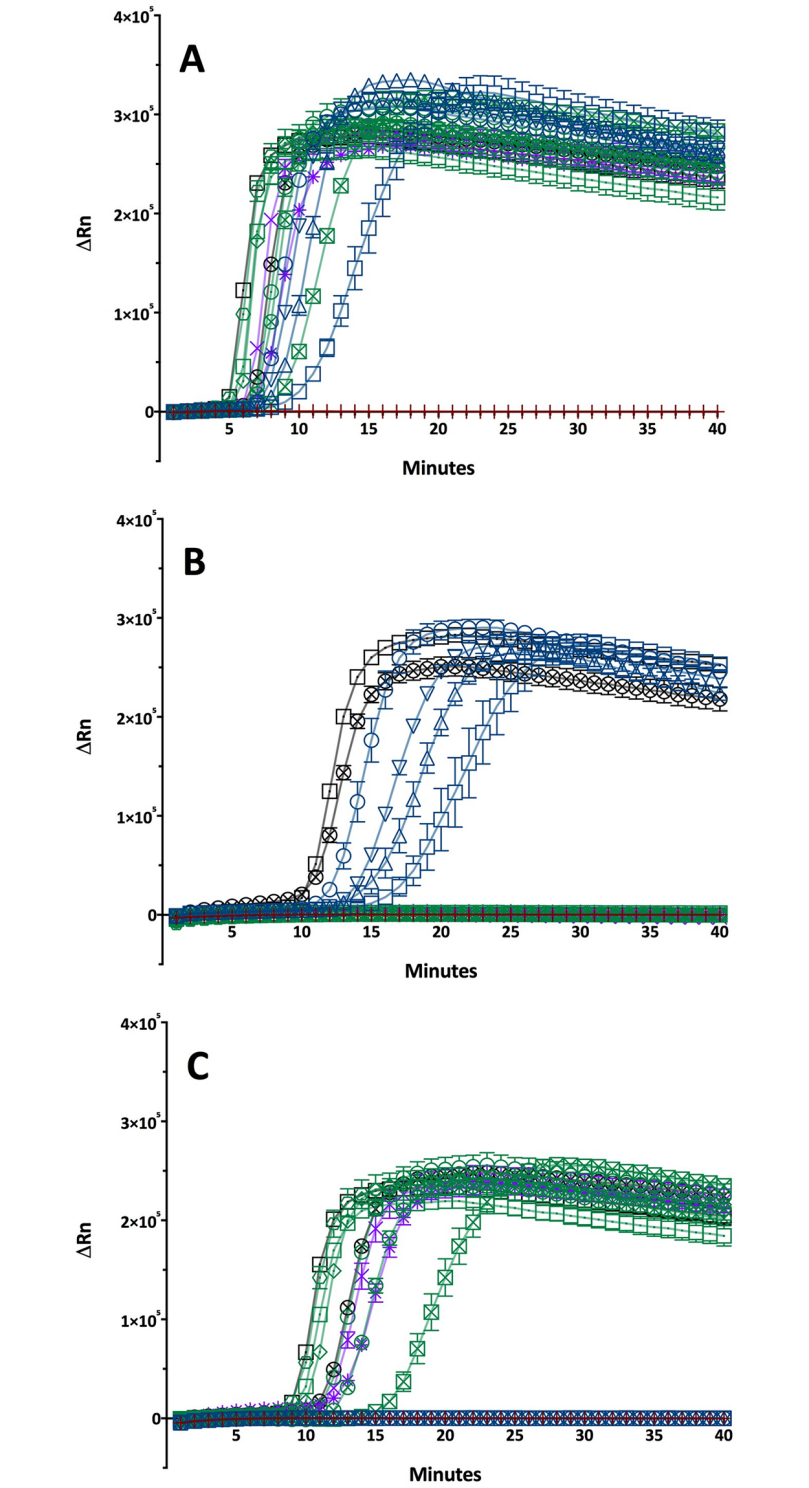
Performance characteristics of the three LAMP assays. (A) LAMP targeting the *polA* gene. All *TP* strains become positive. (B) LAMP assay targeting the *tprL* locus, which results in amplification of *TPA* and *TEN* strains. (C) Only *TPE* and *TEN* strains generate positive results in the LAMP assay targeting the TP_0619 locus. Note that the strain material in the three assays had different *TP* copy numbers as indicated in S1 Table. Data points are presented as mean±SEM values. Red cross = Negative control; *TPA* strains (blue) up-pointing triangle = Mexico A, down-pointing triangle = SS14, circle = Nichols, square = Seattle 81–4; *TPE* strains (green) square = Gauthier, square turned = Sei Geringing K403, hexagon = Kampung Dalan K363, circle = Samoa D, circle with cross = CDC-1, square with cross CDC-2; *TPE* simian strains (purple) cross = Fribourg-Blanc, star = RNP; *TEN* strains (black) square = Bosnia A, circle with cross = Iraq B.

The LAMP primer set ‘*TP*’ targets the polymerase I (*polA*) gene (TP_0105) of *TP*. The locus is highly specific for all *TP* subspecies [22] and has only one orthologue in the lagomorph infecting *Treponema paraluisleporidarum* ecovar *Cuniculus*. The latter is not capable of infecting humans [23, 24]. This locus therefore allows the reaction to become positive for DNA of any known *TPA*, *TPE*, or *TEN* strain (Fig 1A).

LAMP primer set ‘*TPA*/*TEN*’ targets the *tprL* gene (TP_1031) of *TP*. At this locus, a 278-bp long deletion exists that distinguishes known human *TPA* and *TEN* strains from human and simian *TPE* strains [25, S1 Fig]. This primer set was specifically designed to bind within the deletion part, which creates the specificity for *TPA* and *TEN* strains (Fig 1B).

LAMP primer set ‘*TPE*/*TEN’* targets the *T*. *pallidum* TP_0619 gene, which has recently been investigated in a multilocus-typing study on *TPE* strains [26]. This locus has a 179-bp long sequence part that distinguishes known human and NHP *TPE* as well as *TEN* strains from *TPA* human strains (Fig 1C, S2 Fig). Primer sequence data of all three LAMP primer sets are listed in Table 1.

### Quantification of strain material

All *TP* strains used in this study were quantified using an established [27] but slightly modified TaqMan PCR (qPCR) targeting the *polA* gene. A dilution series of a plasmid containing the target amplicon was used as a standard curve from 10E+7 to 10E+0 copy numbers. Briefly, each reaction volumes contained 10.0 μl TaqMan Universal Mastermix II (no Uracil N-glycosylase, Applied Biosystems), 1.8 μl each 10 μM primer and probe, 3.6 μl molecular grade water (RNase-free; Qiagen), and one microliter of the target DNA. Samples were quantified using a StepOne Plus Real-time system with the following temperature steps: 50°C for two minutes, 95°C for ten minutes, followed by 50 cycles of 95°C for 15 seconds and 60°C for one minute. At the end of each cycle, fluorescence was measured. All samples and standards were run as triplicates.

### Data analyses and statistics

LAMP performance as well as qPCR data were retrieved from the StepOnePlus Real-Time PCR System and extracted as RAW data into Excel sheets utilizing the StepOne Software v2.3 (Life Technologies). Statistical analyses were performed with Prism 7.0 (GraphPad Software). In LAMP dilution series with low copy numbers (≤10E+2) and in qPCR data, single replicate outliers were excluded.

## Results

### LAMP assays are highly specific for *T*. *pallidum* target DNA

The LAMP assay targeting the *polA* gene was positive for all tested *TP* strain samples including the four *TPA*, six human *TPE*, two simian *TPE*, and the two human *TEN* strains (Fig 1A, S1 Table). The *tprL* targeting LAMP was positive for all tested *TPA* and *TEN* strains, while human and NHP *TPE* strains did not amplify (Fig 1B). The LAMP assay that uses a part of the TP_0619 gene generated positive results for all *TPE* strains including simian *TPE* strains as well as the two human *TEN* strains (Fig 1C). The onset of exponential fluorescence increase (ΔRn) started reproducibly between 5 min and 15 min incubation time (Fig 1A–1C). Melting curves for each LAMP assay are shown in S3 Fig. All curves were of appropriate shape and without any additional peaks indicative for unwanted side products of primer dimers.

### Limits of detection are suitable for clinical samples

**Fig 2:**
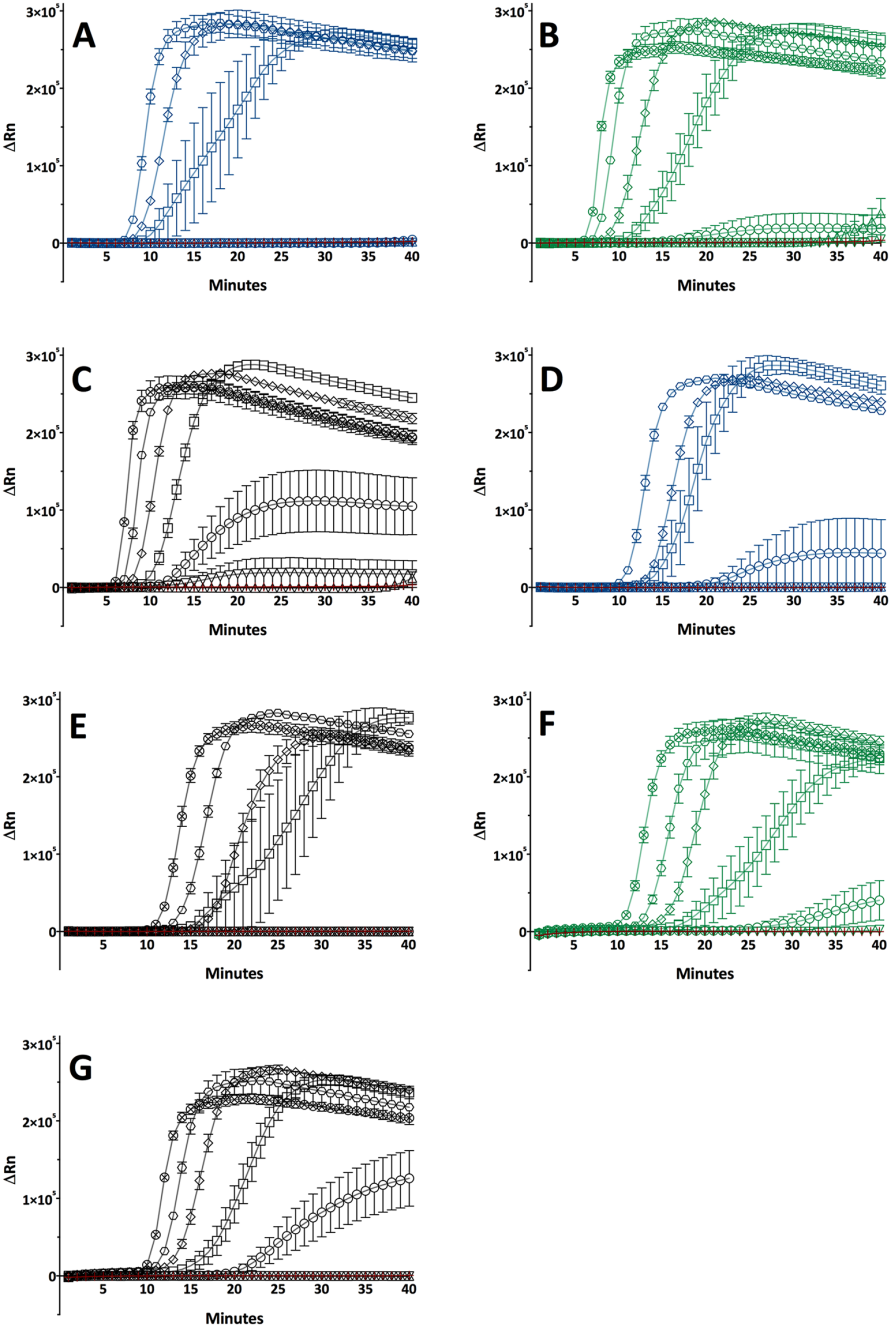
Detection limits of LAMP assays. (A-C) *TP* LAMP assay involving (A) *TPA* strain Nichols with copy numbers 10E+5 to 10E+0, (B) *TPE* strain Gauthier tested in a range of 10E+6 to 10E+0 copies, and (C) *TEN* strain Bosnia A in 10E+6 to 10E+0 copy numbers. (D-E) *TPA* LAMP assay run with *TPA* strain Nichols from 10E+5 to 10E+0 copy numbers, and the same LAMP assay run with (E) *TEN* strain Bosnia A in copy numbers ranging from 10E+6 to 10E+0. (F-G) *TPE*/*TEN* LAMP assay involving (E) *TPE* strain Gauthier tested in 10E+6 to 10E+0 copy numbers, and (F) *TEN* strain Bosnia A tested in 10E+6 to 10E+0 copies. Red cross = Negative control, *TPA* strain Nichols (blue), *TPE* strain Gauthier (green), *TEN* strain Bosnia A (black); symbols represent copy numbers: circle with cross = 10E+6, hexagon = 10E+5, square turned = 10E+4, square = 10E+3, circle = 10E+2, down-pointing triangle = 10E+1, up-pointing triangle = 10E+0.

Analytic limits of detection were assessed as demonstrated in several published studies [28–30]. The LAMP assay that targets the *polA* locus amplifies all *TP* strains but differed slightly in its detection limit across the different *TP* subspecies. While the *TPA* strain Nichols failed to amplify between 10E+3 and 10E+2 copies (Fig 2A), the *TPE* strain Gauthier showed a non-exponential increase in fluorescence at 10E+2 copies (Fig 2B). *TEN* strain Bosnia A failed to exponentially amplify at 10E+1 copies (Fig 2C; Table 2). The LAMP targeting the *tprL* locus had a detection limit of 10E+2 copies for Nichols (Fig 2D) and 10E+3 for *TEN* strain Bosnia A (Fig 2E; Table 2).

**Table 2:** Detection limits for the three LAMP assays. Corresponding graphs can be found in Fig 2. + = exponential amplification, (+) = no exponential amplification,— = no amplification.

	Gene	*TP*	Dilution step (total *TP* copy number per reaction)						
LAMP	Locus	Strain	10E+6	10E+5	10E+4	10E+3	10E+2	10E+1	10E+0
*TP*	*polA* (TP_0105)	Nichols	n/a	+	+	+	-	-	-
		Gauthier	+	+	+	+	(+)	-	-
		Bosnia A	+	+	+	+	+	(+)	-
*TPA*/*TEN*	*tprL* (TP_1031)	Nichols	n/a	+	+	+	+	-	-
		Bosnia A	+	+	+	+	-	-	-
*TPE*/*TEN*	TP_0619	Gauthier	+	+	+	+	(+)	-	-
		Bosnia A	+	+	+	+	+	-	-

The LAMP assay that utilizes the TP_0619 locus amplified *TPE* (strain Gauthier) and *TEN* (strain Bosnia A) DNA until a total copy number of 10E+2 copies was reached (Fig 2F and 2G; Table 2).

## Discussion

### Suitability of the selected gene targets for *T*. *pallidum* subsp. discriminating LAMP assays

In many areas where endemic treponematoses occur, syphilis can also be found at meaningful prevalence rates (e.g., Ghana 3.7% [31], Papua New Guinea 7.9% (men)-12.9% (women) [32]). While this is a problem for the serological based diagnosis of yaws in the presence of etiologically unrelated skin ulcers, it is not an issue for LAMP assays, which specifically target the DNA of the pathogen. The *TPE*/*TEN* LAMP was able to reliably discriminate yaws and simian *TPE* infection from infection with syphilis causing strains. It will, however, not discriminate yaws-causing strains from those known to cause bejel (*TEN* strains). While in theory this could be a problem, bejel is a disease found in the dry areas of Sahelian Africa and Saudia Arabia and thus its spatial distribution does not overlap with yaws reporting countries in Western and Central Africa, Southeast Asia, and the Pacific Islands [33]. In cases where a clear differentiation between yaws and bejel infection is important, the combination of the LAMP targeting *tprL1* and TP_0619 will enable the distinction of both subspecies since only *TEN* strains will amplify in both assays. In the future, either *TEN* specific assays or a LAMP multiplex assay can be designed [34]. The latter, however, would require a fluorescence measuring device and thus may restricts the use in remote tropical health care facilities. Our study used the StepOnePlus Real-Time PCR System, but all LAMP assays described in this study can be run equally well on a portable system (e.g., ESEQuant TS2, Qiagen) that allows easy transportation and use under field conditions. In low-income clinical settings, it would even be possible to detect amplification by the naked eye through the detection of turbidity generated by the precipitation of magnesium pyrophosphate or through the addition of calcein, a fluorescent metal indicator [35]. Lyophilization allows for ambient storage of formulated LAMP reagents [36].

As indicated in the methods, all three gene targets that were selected for the LAMP assays are highly specific for the human and NHPs specific pathogenic *TP*, but also the lagomorph infecting *T*. *paraluisleporidarum* ecovar *Cuniculus* and *Lepus*, respectively. However, lagomorph infecting treponemes are not capable of infecting humans [23, 24] and most probably also NHPs. False positive test results due to infection with non-*TP* bacteria are therefore unlikely. In light of a recently published report on failure of qPCR due to variations in primer binding sites [37], it should be noted that the number of published genomes, in particular non-draft genomes, in any of the *TP* subspecies is low. At this stage, a general statement on genome variability at the selected gene target sites is therefore not possible. However, based on our research, which included representatives of the full range of published *TP* genomes (Table 1, S1 and S2 Figs), the relevant primer binding sites are conserved across the different subspecies and strains.

### LAMP as a rapid and reliable discrimination tool

It has been proposed that yaws eradication in humans is possible through total community treatment in combination with subsequent total target treatment [38]. Rapid and reliable identification of yaws infection is important because successful global eradication requires an infinite zero-case scenario. In the first years after eradication has been declared in humans, it might well happen that few cases reemerge from yet unidentified relapsing latent yaws cases as well as there is a theoretical change that sporadic spillover from infected wild NHPs in Africa occurs. Either way, an available molecular test such as a LAMP assay could effectively and timely identify new cases from etiologically unrelated skin ulcers at the very beginning and could help to prevent yaws from re-emerging in areas where PCR machines and expensive laboratory equipment are not available. The analytic limits of detection for all three LAMP assays were around 10E+2 copies per reaction (Table 2), which is sufficient for clinical samples from human primary and secondary syphilis infection [39]. The same numbers can be expected for human yaws samples. Furthermore, the amount of *TP* in chronically infected monkeys also falls within the detection range of the *TPE*/*TEN* LAMP [27].

### Application for epidemiological studies in nonhuman primates and one health

NHP *TPE* strains have been discussed as a possible source for human yaws infection in Africa [13]. The identification of NHP populations that harbor the pathogen, not only in Africa but also Asia [12], must be considered an important research priority [4]. Post-treatment surveillance needs to focus in particular on areas where NHPs and humans are in close contact. The *TPE*/*TEN* LAMP performance of the NHP samples (strain Fribourg-Blanc and DNA extracted from a clinical sample of a baboon at Ruaha National Park in Tanzania (RNP)) that were included into this study were similar to the results obtained for the human yaws-causing strains (Fig 1C). This is not surprising, given the fact that NHP *TPE* strains are genetically and functionally highly similar to human yaws causing strains [14, 15]. However, the full diversity of NHP infecting *TP* is unknown and it is possible that monkeys from Sahelian Africa and Saudia Arabia may carry *TEN* strains. In this case, the *TPE*/*TEN* LAMP assay would become positive. Due to the fact that currently all naturally occurring NHP infections with *TP* should be whole genome sequenced to fuel our understanding on yaws epidemiology and evolution, the *TPE*/*TEN* LAMP assay result would be more of academic than practical interest. The whole genome data derived from simian isolates would reveal the subspecies status of the isolate.

**Fig 3:**
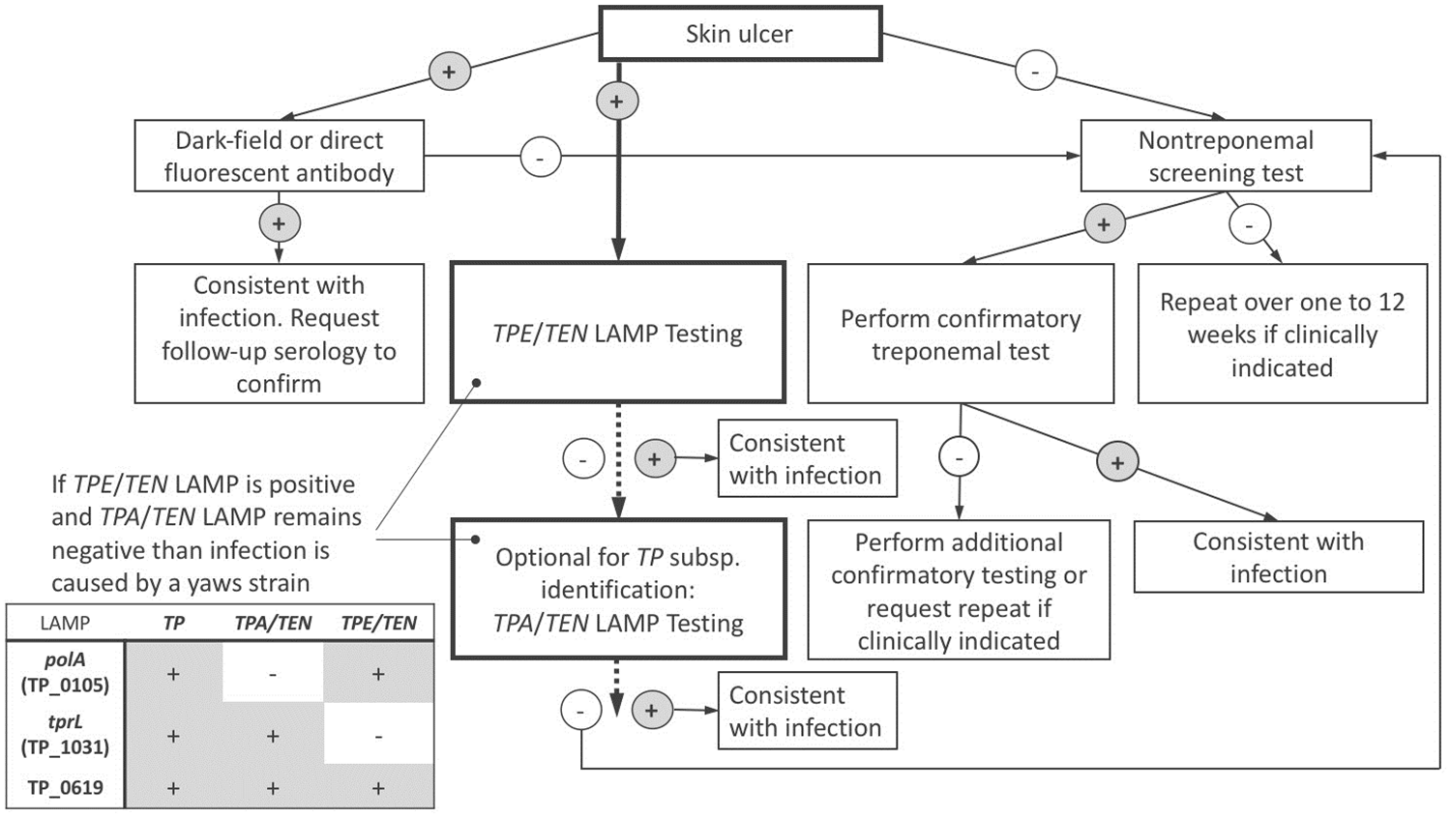
LAMP assays will simplify the existing test algorithm for human treponematoses. The test algorithm proposed by Ratnam 2005 [42] is shown in thin lines. The new LAMP assays for diagnosis of yaws is shown in bold lines and will simplify and accelerate yaws diagnosis. Furthermore, it enables the clinician to rapidly discriminate *TPE* infection from infection with other *TP* subspecies or pathogens.

In humans, infections with all *TP* subspecies have reported potential to cause atypical clinical manifestations. A striking example is the frequent syphilis-like manifestations associated with *TEN* strains [40,41]. A rapid, highly sensitive and specific LAMP assay would therefore contribute to the identification of atypical clinical manifestations caused by *TP*. It would further help to identify possible NHP-to-human infection in countries like Tanzania, where human yaws has not been reported since decades. Syphilis screening programs in Tanzania would currently not detect possible NHP-to-human transmission events, since serological tests cannot discriminate between the *TPA* and *TPE* infection. Our target selection for LAMP assays that discriminate infection with *TP* from other causes of skin ulcers, represents a basis for the implementation of a One Health approach in yaws eradication and its post-eradication surveillance. Fig 3 illustrates the proposed new way of diagnosing *TPE* infection in humans. The new LAMP assays would simplify and accelerate yaws diagnosis. We note here that we have reached proof of concept for the suitability of the described gene targets, but further validation in a statistically adequate number of clinical samples is necessary to achieve confidence of the LAMP assays to be used in a non-research environment.

### Conclusion

The selected gene targets are suitable for the diagnosis and discrimination of all three *TP* subspecies, which is currently not possible using clinical signs of infection in combination with serology. The next step must be to conduct tests to evaluate the sensitivity and specificity of the newly created assays in various clinical samples that originate from humans and NHPs. The designed LAMP assays do not require expensive laboratory equipment and can be run in virtually any clinical setting. Results are available within a few minutes and thus outrun the existing methods, which require reasonable laboratory infrastructure [4].

## Supporting information

S1 Fig *tprL* alignment showing the sequence differences between TPA/TEN and TPE strains. Nucleotide position 1 in the alignment refers to nucleotide position 1,125587 in the TPA str. Nichols genome (CP004010) and nucleotide position 410 to 1,125,993, respectively. Green annotations on the consensus sequence indicate the outer LAMP primer binding sites F3 and B3 (Table 1). GenBank accession numbers for TPA: CP010561 str. Nichols Houston clone J, CP010560 str. Nichols Houston clone E, CP004010 str. Nichols, AE000520 str. Nichols, HM 585246 str. DAL-1, CP003115 str. DAL-1, CP010558 str. Chicago, CP001752 str. Chicago, CP010422 str. Seattle Nichols, CP003679 str. Sea 81–4, CP010559 str. CDC-A, CP004011 str. SS14, CP000805 str. SS14, CP016066 str. PT_SIF1278, CP016057 str. PT_SIF1142, CP016056 str. PT_SIF121140, CP01050 str. PT_SIF0954, CP016049 str. PT_SIF0908, CP016045 str. PT_SIF0697, CP016055 str. PT_SIF1135, CP016053 str. PT_SIF1063, CP016069 str. PT_SIF1348, CP016068 str. PT_SIF1299, CP016067 str. PT_SIF1280, CP016065 str. PT_SIF1261, CP016064 str. PT_SIF1252, CP016063 str. PT_SIF1242, CP016062 str. PT_SIF1200, CP0116061 str. PT_SIF1196, CP016060 str. PT_SIF1183, CP016058 str. PT_SIF1156, CP016051 str. PT_SIF1002, CP016048 str. PT_SIF0877, CP016047 str. PT_SIF0857, CP016046 str. PT_SIF0751, CP016059 str. PT_SIF1167, CP016052 str. PT_SIF1020, CP016054 str. PT_SIF1127, CP010562 str. UW074B, CP010563 str. UW189B, CP010565 str. UW254B, CP010564 str. UW228B, CP010566 str. UW391B, CP015162 str. Amoy, NC_018722 str. MexA, CP003064 str. MexA, HM585253 str. MexA, TEN: CP007548 str. BosA, TPE: JX079830 str. Gauthier, HM585235.1 str. Gauthier, CP002375.1 str. CDC-2, JX079832 str. CDC-2, CP020365 str. Ghana-051, CP020366 str. CDC-2575, CP002374.1 str. SamoaD, JX079831 str. SamoaD, ERR1470330, ERR1470331, ERR1470334, ERR1470338, ERR1470343, ERR1470344, JX079833 str. Fribourg-Blanc, CP003902.1 str. Fribourg-Blanc.
(DOCX)

S2 Fig TP_0619 alignment showing the sequence differences between TPA and TPE/TEN strains. Nucleotide position 1 in the alignment refers to nucleotide position 671,979 in the TPA str. Nichols genome (CP004010) and nucleotide position 239 to 672,216, respectively. Green annotations on the consensus sequence indicate the outer LAMP primer binding sites F3 and B3 (Table 1). GenBank accession numbers for TPA: CP003115 str. DAL-1, CP000805 str. SS14, CP004011 str. SS14, CP001752 str. Chicago, CP003064 str. MexA, CP004010 str. Nichols, AE000520 str. Nichols, CP003679 str. Sea81-4, CP010559 str. CDC A, CP010558 str. Chicago, CP010560 str. Nichols Houston, CP010561 str. Nichols Houston, CP016045 str. PT_SIF0697, CP016046 str. PT_SIF0751, CP016047 str. PT_SIF0857, CP016048 str. PT_SIF0877, CP016049 str. PT_SIF0908, CP016050 str. PT_SIF0954, CP016051 str. PT_SIF1002, CP016052 str. PT_SIF1020, CP016053 CP016054 str. PT_SIF1127, CP016055 str. PT_SIF1135, CP016056 str. PT_SIF1140, CP016057 str. PT_SIF1142, CP016058 str. PT_SIF1156, CP016059 str. PT_SIF1167, CP016060 str. PT_SIF1183, CP016061 str. PT_SIF1196, CP016062 str. PT_SIF1200, CP016063 str. PT_SIF1242, CP016064 str. PT_SIF1252, CP016065 str. PT_SIF1261, CP016066 str. PT_SIF1278, CP016067 str. PT_SIF1280, CP016068 str. PT_SIF1299, CP016069 str. PT_SIF1348, CP010422 str. Seattle Nichols, CP10562 str. UW074B, CP10563 str. UW189B, CP10564 str. UW228B, CP10565 str. UW254B, CP10566 str. UW391B, TEN: CP007548 str. BosA, KY120834.1 str. IraqB, TPE: CP002375 str. CDC2, CP002376 str. Gauthier, CP002374 str. SamoaD, CP020366 str. CDC2575, CP020365 str. Ghana-051, CP003902 str. Fribourg-Blanc, MG573304 str. RuahaNP-1 (6RUM2090716).
(DOCX)

S3 Fig Melting curves of the different TP LAMP assays. Melting curves were of appropriate shape and without any additional peaks indicative for unwanted side products or primer dimers. LAMP targeting (A) the *polA* gene, (B) the *tprL* locus, and (C) the TP_0619 locus.
(DOCX)

S1 Table Details and further reference on *T*. *pallidum* strains included into the study. RNP = Ruaha National Park.
(DOCX)

S1 References (DOCX)
