# Supplementary material for: Gene target selection for loop-mediated isothermal amplification for rapid discrimination of Treponema pallidum subspecies
Source: PLoS Negl Trop Dis. 2018 Apr 12;12(4):e0006396. doi: 10.1371/journal.pntd.0006396 (PMC5978989; doi:10.1371/journal.pntd.0006396)
Supplement: S1 Fig — Nucleotide position 1 in the alignment refers to nucleotide position 1,125587 in the TPA str. Nichols genome (CP004010) and nucleotide position 410 to 1,125,993, respectively. Green annotations on the consensus sequence indicate the outer LAMP primer binding sites F3 and B3 (Table 1). GenBank accession numbers for TPA: CP010561 str. Nichols Houston clone J, CP010560 str. Nichols Houston clone E, CP004010 str. Nichols, AE000520 str. Nichols, HM 585246 str. DAL-1, CP003115 str. DAL-1, CP010558 str. Chicago, CP001752 str. Chicago, CP010422 str. Seattle Nichols, CP003679 str. Sea 81–4, CP010559 str. CDC-A, CP004011 str. SS14, CP000805 str. SS14, CP016066 str. PT_SIF1278, CP016057 str. PT_SIF1142, CP016056 str. PT_SIF121140, CP01050 str. PT_SIF0954, CP016049 str. PT_SIF0908, CP016045 str. PT_SIF0697, CP016055 str. PT_SIF1135, CP016053 str. PT_SIF1063, CP016069 str. PT_SIF1348, CP016068 str. PT_SIF1299, CP016067 str. PT_SIF1280, CP016065 str. PT_SIF1261, CP016064 str. PT_SIF1252, CP016063 str. PT_SIF1242, CP016062 str. PT_SIF1200, CP0116061 str. PT_SIF1196, CP016060 str. PT_SIF1183, CP016058 str. PT_SIF1156, CP016051 str. PT_SIF1002, CP016048 str. PT_SIF0877, CP016047 str. PT_SIF0857, CP016046 str. PT_SIF0751, CP016059 str. PT_SIF1167, CP016052 str. PT_SIF1020, CP016054 str. PT_SIF1127, CP010562 str. UW074B, CP010563 str. UW189B, CP010565 str. UW254B, CP010564 str. UW228B, CP010566 str. UW391B, CP015162 str. Amoy, NC_018722 str. MexA, CP003064 str. MexA, HM585253 str. MexA, TEN: CP007548 str. BosA, TPE: JX079830 str. Gauthier, HM585235.1 str. Gauthier, CP002375.1 str. CDC-2, JX079832 str. CDC-2, CP020365 str. Ghana-051, CP020366 str. CDC-2575, CP002374.1 str. SamoaD, JX079831 str. SamoaD, ERR1470330, ERR1470331, ERR1470334, ERR1470338, ERR1470343, ERR1470344, JX079833 str. Fribourg-Blanc, CP003902.1 str. Fribourg-Blanc. (DOCX) [file pntd.0006396.s001.docx]

**Fig S1. *tprL* alignment showing the sequence differences between TPA/TEN and TPE strains.** Nucleotide position 1 in the alignment refers to nucleotide position 1,125587 in the TPA str. Nichols genome (CP004010) and nucleotide position 410 to 1,125,993, respectively. Green annotations on the consensus sequence indicate the outer LAMP primer binding sites F3 and B3 (Table 1). GenBank accession numbers for TPA: CP010561 str. Nichols Houston clone J, CP010560 str. Nichols Houston clone E, CP004010 str. Nichols, AE000520 str. Nichols, HM 585246 str. DAL-1, CP003115 str. DAL-1, CP010558 str. Chicago, CP001752 str. Chicago, CP010422 str. Seattle Nichols, CP003679 str. Sea 81-4, CP010559 str. CDC-A, CP004011 str. SS14, CP000805 str. SS14, CP016066 str. PT_SIF1278, CP016057 str. PT_SIF1142, CP016056 str. PT_SIF121140, CP01050 str. PT_SIF0954, CP016049 str. PT_SIF0908, CP016045 str. PT_SIF0697, CP016055 str. PT_SIF1135, CP016053 str. PT_SIF1063, CP016069 str. PT_SIF1348, CP016068 str. PT_SIF1299, CP016067 str. PT_SIF1280, CP016065 str. PT_SIF1261, CP016064 str. PT_SIF1252, CP016063 str. PT_SIF1242, CP016062 str. PT_SIF1200, CP0116061 str. PT_SIF1196, CP016060 str. PT_SIF1183, CP016058 str. PT_SIF1156, CP016051 str. PT_SIF1002, CP016048 str. PT_SIF0877, CP016047 str. PT_SIF0857, CP016046 str. PT_SIF0751, CP016059 str. PT_SIF1167, CP016052 str. PT_SIF1020, CP016054 str. PT_SIF1127, CP010562 str. UW074B, CP010563 str. UW189B, CP010565 str. UW254B, CP010564 str. UW228B, CP010566 str. UW391B, CP015162 str. Amoy, NC_018722 str. MexA, CP003064 str. MexA, HM585253 str. MexA, TEN: CP007548 str. BosA, TPE: JX079830 str. Gauthier, HM585235.1 str. Gauthier, CP002375.1 str. CDC-2, JX079832 str. CDC-2, CP020365 str. Ghana-051, CP020366 str. CDC-2575, CP002374.1 str. SamoaD, JX079831 str. SamoaD, ERR1470330, ERR1470331, ERR1470334, ERR1470338, ERR1470343, ERR1470344, JX079833 str. Fribourg-Blanc, CP003902.1 str. Fribourg-Blanc.
