# Supplementary material for: Gene target selection for loop-mediated isothermal amplification for rapid discrimination of Treponema pallidum subspecies
Source: PLoS Negl Trop Dis. 2018 Apr 12;12(4):e0006396. doi: 10.1371/journal.pntd.0006396 (PMC5978989; doi:10.1371/journal.pntd.0006396)
Supplement: S2 Fig — Nucleotide position 1 in the alignment refers to nucleotide position 671,979 in the TPA str. Nichols genome (CP004010) and nucleotide position 239 to 672,216, respectively. Green annotations on the consensus sequence indicate the outer LAMP primer binding sites F3 and B3 (Table 1). GenBank accession numbers for TPA: CP003115 str. DAL-1, CP000805 str. SS14, CP004011 str. SS14, CP001752 str. Chicago, CP003064 str. MexA, CP004010 str. Nichols, AE000520 str. Nichols, CP003679 str. Sea81-4, CP010559 str. CDC A, CP010558 str. Chicago, CP010560 str. Nichols Houston, CP010561 str. Nichols Houston, CP016045 str. PT_SIF0697, CP016046 str. PT_SIF0751, CP016047 str. PT_SIF0857, CP016048 str. PT_SIF0877, CP016049 str. PT_SIF0908, CP016050 str. PT_SIF0954, CP016051 str. PT_SIF1002, CP016052 str. PT_SIF1020, CP016053 CP016054 str. PT_SIF1127, CP016055 str. PT_SIF1135, CP016056 str. PT_SIF1140, CP016057 str. PT_SIF1142, CP016058 str. PT_SIF1156, CP016059 str. PT_SIF1167, CP016060 str. PT_SIF1183, CP016061 str. PT_SIF1196, CP016062 str. PT_SIF1200, CP016063 str. PT_SIF1242, CP016064 str. PT_SIF1252, CP016065 str. PT_SIF1261, CP016066 str. PT_SIF1278, CP016067 str. PT_SIF1280, CP016068 str. PT_SIF1299, CP016069 str. PT_SIF1348, CP010422 str. Seattle Nichols, CP10562 str. UW074B, CP10563 str. UW189B, CP10564 str. UW228B, CP10565 str. UW254B, CP10566 str. UW391B, TEN: CP007548 str. BosA, KY120834.1 str. IraqB, TPE: CP002375 str. CDC2, CP002376 str. Gauthier, CP002374 str. SamoaD, CP020366 str. CDC2575, CP020365 str. Ghana-051, CP003902 str. Fribourg-Blanc, MG573304 str. RuahaNP-1 (6RUM2090716). (DOCX) [file pntd.0006396.s002.docx]

**Fig S2. TP_0619 alignment showing the sequence differences between TPA and TPE/TEN strains.** Nucleotide position 1 in the alignment refers to nucleotide position 671,979 in the TPA str. Nichols genome (CP004010) and nucleotide position 239 to 672,216, respectively. Green annotations on the consensus sequence indicate the outer LAMP primer binding sites F3 and B3 (Table 1). GenBank accession numbers for TPA: CP003115 str. DAL-1, CP000805 str. SS14, CP004011 str. SS14, CP001752 str. Chicago, CP003064 str. MexA, CP004010 str. Nichols, AE000520 str. Nichols, CP003679 str. Sea81-4, CP010559 str. CDC A, CP010558 str. Chicago, CP010560 str. Nichols Houston, CP010561 str. Nichols Houston, CP016045 str. PT_SIF0697, CP016046 str. PT_SIF0751, CP016047 str. PT_SIF0857, CP016048 str. PT_SIF0877, CP016049 str. PT_SIF0908, CP016050 str. PT_SIF0954, CP016051 str. PT_SIF1002, CP016052 str. PT_SIF1020, CP016053 CP016054 str. PT_SIF1127, CP016055 str. PT_SIF1135, CP016056 str. PT_SIF1140, CP016057 str. PT_SIF1142, CP016058 str. PT_SIF1156, CP016059 str. PT_SIF1167, CP016060 str. PT_SIF1183, CP016061 str. PT_SIF1196, CP016062 str. PT_SIF1200, CP016063 str. PT_SIF1242, CP016064 str. PT_SIF1252, CP016065 str. PT_SIF1261, CP016066 str. PT_SIF1278, CP016067 str. PT_SIF1280, CP016068 str. PT_SIF1299, CP016069 str. PT_SIF1348, CP010422 str. Seattle Nichols, CP10562 str. UW074B, CP10563 str. UW189B, CP10564 str. UW228B, CP10565 str. UW254B, CP10566 str. UW391B, TEN: CP007548 str. BosA, KY120834.1 str. IraqB, TPE: CP002375 str. CDC2, CP002376 str. Gauthier, CP002374 str. SamoaD, CP020366 str. CDC2575, CP020365 str. Ghana-051, CP003902 str. Fribourg-Blanc, MG573304 str. RuahaNP-1 (6RUM2090716).
