# Supplementary material for: Gene target selection for loop-mediated isothermal amplification for rapid discrimination of Treponema pallidum subspecies
Source: PLoS Negl Trop Dis. 2018 Apr 12;12(4):e0006396. doi: 10.1371/journal.pntd.0006396 (PMC5978989; doi:10.1371/journal.pntd.0006396)
Supplement: S3 Fig — Melting curves were of appropriate shape and without any additional peaks indicative for unwanted side products or primer dimers. LAMP targeting (A) the polA gene, (B) the tprL locus, and (C) the TP_0619 locus. (DOCX) [file pntd.0006396.s003.docx]

**
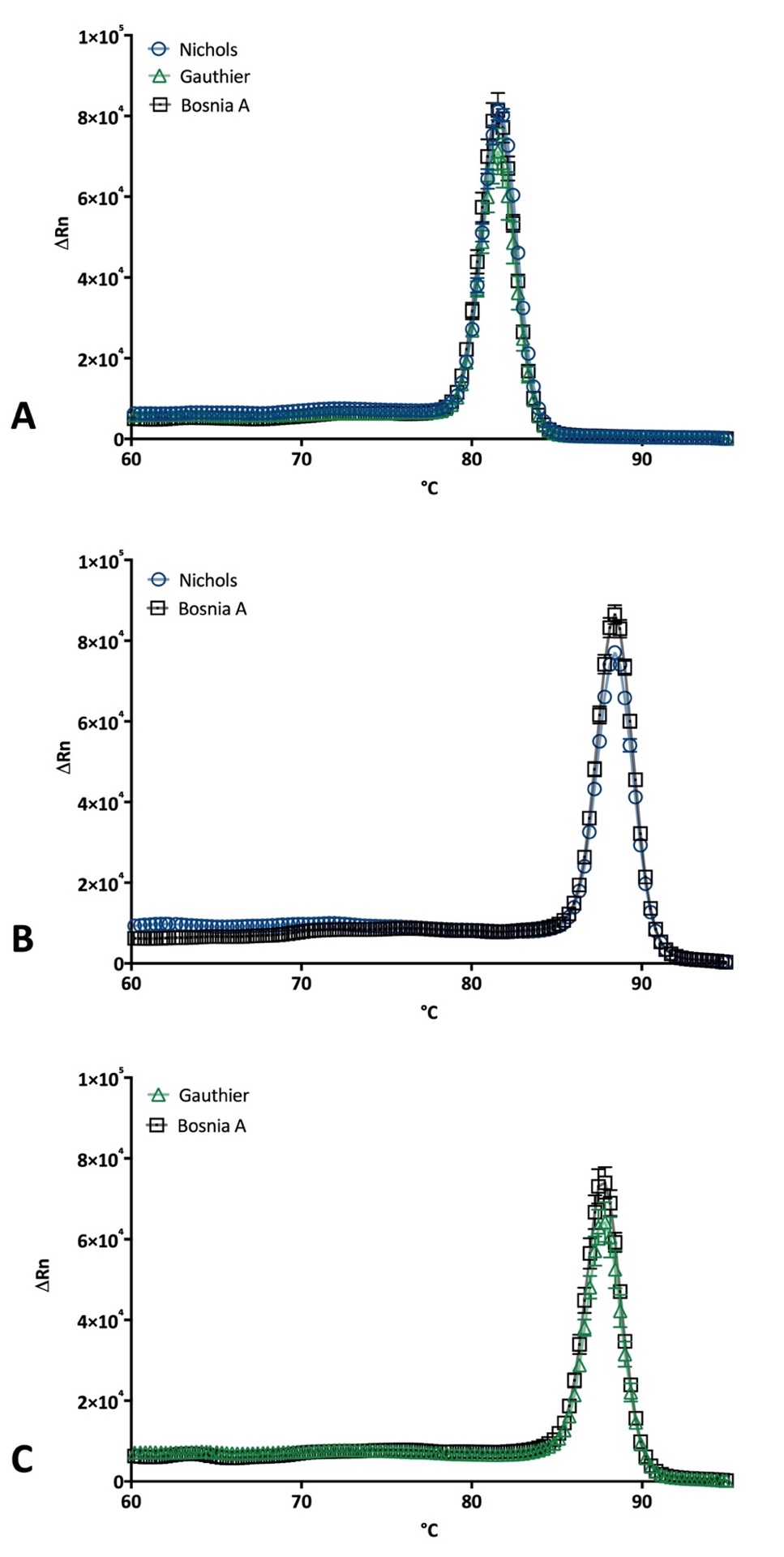
**

**Fig S3. Melting curves of the different TP LAMP assays.** Melting curves were of appropriate shape and without any additional peaks indicative for unwanted side products or primer dimers. LAMP targeting (A) the *polA* gene, (B) the *tprL* locus, and (C) the TP_0619 locus.
