# Supplementary material for: Gene target selection for loop-mediated isothermal amplification for rapid discrimination of Treponema pallidum subspecies
Source: PLoS Negl Trop Dis. 2018 Apr 12;12(4):e0006396. doi: 10.1371/journal.pntd.0006396 (PMC5978989; doi:10.1371/journal.pntd.0006396)
Supplement: S1 Table — RNP = Ruaha National Park. (DOCX) [file pntd.0006396.s004.docx]

**Supporting information**

**Table S1. Details and further reference on *T. pallidum* strains included into the study.** RNP = Ruaha National Park

| **TP subspecies** | **Strain** | **Origin** | **Laboratory or Wildtype** | **Copy number/µl** | **Reference^$^ for strain classification** |
| --- | --- | --- | --- | --- | --- |
| *pallidum* | Mexico A | Human | Laboratory | 4.48E+4 | [SR1] |
|  | SS14 | Human | Laboratory | 4.98E+5 | [SR1] |
|  | Nichols | Human | Laboratory | 1.32E+5 | [SR1] |
|  | Seattle 81-4 | Human | Laboratory | 7.08E+3 | [SR1] |
| *pertenue* | Gauthier | Human | Laboratory | 1.28E+7 | [SR1] |
|  | Sei Geringging K403 | Human | Laboratory | 1.95E+7 | [SR2] |
|  | Kampung Dalan K363 | Human | Laboratory | 1.70E+8 | [SR2] |
|  | Samoa D | Human | Laboratory | 1.00E+6 | [SR1] |
|  | CDC-1 | Human | Laboratory | 4.33E+5 | [SR3] |
|  | CDC-2 | Human | Laboratory | 2.82E+4 | [SR1] |
| Formally unclassified | Fribourg-Blanc | *Papio papio* | Laboratory | 2.72E+6 | [15] |
| Formally unclassified | RNP | *Papio anubis*  (6RUM2090716) | Wild | 1.27E+5 |  |
| *endemicum* | Bosnia A | Human | Laboratory | 1.95E+7 | [SR5] |
|  | Iraq B | Human | Laboratory | 3.42E+5 | [SR6] |

^$^see References and S1 References
