# Supplementary material for: Gene target selection for loop-mediated isothermal amplification for rapid discrimination of Treponema pallidum subspecies
Source: PLoS Negl Trop Dis. 2018 Apr 12;12(4):e0006396. doi: 10.1371/journal.pntd.0006396 (PMC5978989; doi:10.1371/journal.pntd.0006396)
Supplement: S1 References — (DOCX) [file pntd.0006396.s005.docx]

**Supporting information**

**S1 References**

SR1. Arora N, Schuenemann VJ, Jager G, Peltzer A, Seitz A, Herbig A, et al. Origin of modern syphilis and emergence of a pandemic *Treponema pallidum* cluster. Nat Microbiol. 2016;2:16245. doi: 10.1038/nmicrobiol.2016.245. PubMed PMID: 27918528.

SR2. Mikalová L, Strouhal M, Grillova L, Šmajs D. The molecular typing data of recently identified subtype 11q/j of *Treponema pallidum* subsp. *pallidum* suggest imported case of yaws. Sex Transm Dis. 2014;41(9):552-3. Epub 2014/08/15. doi: 10.1097/OLQ.0000000000000165. PubMed PMID: 25118969.

SR3. Čejková D, Zobaníková M, Pospíšilová P, Strouhal M, Mikalová L, Weinstock GM, et al. Structure of rrn operons in pathogenic non-cultivable treponemes: sequence but not genomic position of intergenic spacers correlates with classification of *Treponema pallidum* and *Treponema paraluiscuniculi* strains. J Med Microbiol. 2013;62(Pt 2):196-207. doi: 10.1099/jmm.0.050658-0. PubMed PMID: 23082031; PubMed Central PMCID: PMC3755535.

SR4. Štaudová B, Strouhal M, Zobaníková M, Čejková D, Fulton LL, Chen L, et al. Whole genome sequence of the *Treponema pallidum* subsp. *endemicum* strain Bosnia A: the genome is related to yaws treponemes but contains few loci similar to syphilis treponemes. PLoS Negl Trop Dis. 2014;8(11):e3261. doi: 10.1371/journal.pntd.0003261. PubMed PMID: 25375929; PubMed Central PMCID: PMCPMC4222731.

SR5. Harper KN, Ocampo PS, Steiner BM, George RW, Silverman MS, Bolotin S, et al. On the origin of the treponematoses: a phylogenetic approach. PLoS Negl Trop Dis. 2008;2(1):e148. Epub 2008/02/01. doi: 10.1371/journal.pntd.0000148. PubMed PMID: 18235852; PubMed Central PMCID: PMC2217670.
